# Role of the Wnt/β-catenin signaling pathway in the development of HCC

**DOI:** 10.3389/fimmu.2025.1691297

**Published:** 2025-10-09

**Authors:** Bo Lin, Mengsen Li

**Affiliations:** ^1^ Key Laboratory of Tropical Translational Medicine of Ministry of Education & Hainan Provincial Key Laboratory of Carcinogenesis and Intervention, School of Basic Medicine and Life Sciences, Hainan Medical University, Haikou, Hainan, China; ^2^ Institution of Tumour, Hainan Medical University, Haikou, Hainan, China

**Keywords:** Wnt/β-catenin pathway, hepatocellular carcinoma, tumour microenvironment, immune exclusion, therapy

## Abstract

Abnormalities in the Wnt/β-catenin pathway promote the development of hepatocellular carcinoma (HCC). Mutations in CTNNB1, which encodes β-catenin, are frequently found in clinical HCC samples, as are loss-of-function mutations in signaling pathway regulators such as axis inhibition protein 1 (Axin1) and adenomatous polyposis coli (APC). The activation of the Wnt/β-catenin pathway synergizes with other oncogenic signal molecules such as c-Met or glypican-3, contributing to HCC development. Furthermore, Wnt/β-catenin pathway activation in the tumour microenvironment (TME) leads to cold tumour and resistance to immunotherapy. In this review, we discuss two models of Wnt/β-catenin signaling activation, role of Wnt/β-catenin signaling pathway in the development of HCC, the association between Wnt/β-catenin pathway and tumour angiogenesis, metastasis, and immune escape in the TME, and the targeting of this signaling pathway for HCC treatment.

## Highlights

- Abnormalities in the Wnt/β-catenin pathway promote the development of hepatocellular carcinoma (HCC), especially through immune exclusion and resistance to immunotherapy.
- A second oncogenic signal, such as c-Met or glypican-3, is required for the Wnt/β-catenin pathway to contribute to HCC development; targeting these oncogenic signal molecules in combination with inhibition of the Wnt/β-catenin signaling promotes tumor regression.
- Therapeutic strategies targeting the Wnt/β-catenin pathway include the use of monoclonal antibodies or small molecule inhibitors to target Wnt ligands and the receptors, the targeting of the CTNNB1 gene or related genes, and inhibition of the interaction between β-catenin and TCF or other nuclear transcriptional regulators.

## 1 Introduction

The Wnt/β-catenin signaling pathway, also known as the canonical Wnt signaling pathway, regulates various cellular functions, including cell proliferation and differentiation, which are essential for organ formation, development, and tissue homeostasis. However, dyregulation of this pathway is involved in the initiation and progression of various cancers, including some solid tumours and hematological malignancies. Recent findings suggest that the Wnt/β-catenin pathway significantly influences cancer-related immune regulation, contributing to immune exclusion, especially as immunotherapy becomes prominent and expands. Wnt inhibitors may have broader applications in cancer immunotherapy (1–3).

In the liver, Wnt/β-catenin signaling pathway plays an essential role in maintaining liver homeostasis, metabolic zonation, and regeneration. However, aberrant activation of this pathway drives hepatocellular carcinoma (HCC) development, indicating its unique regulatory mechanism in HCC progression (4–7). HCC arises from hepatocytes through progressive genomic and epigenomic alterations, with frequent mutations in key components of the Wnt/β-catenin pathway—including CTNNB1 (encoding β-catenin), Axin1/2, and APC (4–8). In this review, we discuss how dysregulation of this pathway contributes to HCC pathogenesis and explore potential targeted therapies.

## 2 The molecular regulatory mechanisms of the Wnt/β-catenin pathway

The Wnt/β-catenin signaling pathway is generally not activated in normal hepatocytes, except during cell regeneration (9–11). However, it can be reactivated under specific pathological conditions, such as cancer. β-Catenin is a crucial molecule in this pathway and acts as a transcriptional coactivator (it is not a classical transcription factor alone, as it lacks DNA-binding domains). Because β-catenin lacks the intrinsic ability to bind DNA, It needs to bind to DNA binding protein of the T-cell factor (TCF)/lymphoid enhancer binding factor (LEF) family and recruiting transcriptional machinery to activate gene expression programs (10).

In normal liver tissues, β-catenin forms adherens junctions between cells on the plasma membrane by linking E-cadherin and the cytoskeleton-associated actin (11). When β-caten in accumulates in the cytoplasm and enters the nucleus, it will activate the Wnt/β-catenin signalling. However, in the absence of extracellular Wnt ligands, the pathway remains inactive. In this condition, β-Catenin is regulated in the cytoplasm by the GSK3β–CK1α–APC–Axin1 complex. This complex, also known as the “destruction complex”, consists of glycogen synthase kinase 3β (GSK3β), casein kinase 1α (CK1α), APC, and Axin1. In normal hepatocytes, the Wnt/β-catenin signaling pathway is OFF due to low or absent Wnt levels caused by feedback inhibition. This occurs because β-catenin is phosphorylated and tagged with ubiquitin by the “destruction complex” and subsequently degraded by proteasomes, which keeps β-catenin levels low in the cytoplasm and prevents its entry into the nucleus to promote target gene expression (4–7, 10, 11).

Exon 3 of CTNNB1 encodes the N-terminal region of the β-catenin protein (amino acid residues 5–80). This critical region contains phosphorylation and ubiquitination sites that regulate the stability of β-catenin protein. In cancer, hotspot mutations frequently occur at residues D32, S33, G34, S37, T41 and S45 within exon 3. These sites play critical roles in the regulated degradation of β-catenin: (1) S45​​ serves as the priming phosphorylation site for CK1α; (2) S33, S37 and T41​​ are subsequent phosphorylation sites targeted by GSK-3β; (3) D32 and G34​​ are necessary for binding to βTrCP (a component of ubiquitin E3 ligase) (12, 13).

In the absence of Wnt signaling, CK1α initiates the degradation cascade by phosphorylating S45. This primes β-catenin for sequential phosphorylation by GSK-3β at S33, S37 and T41. The phosphorylated β-catenin is then recognized and bound by β-TrCP, leading to its polyubiquitination and subsequent proteasomal degradation, thereby maintaining low activity of the Wnt signaling pathway (12, 13).

Hotspot mutations in exon 3 disrupt this precisely ordered phosphorylation and ubiquitination process. Consequently, β-catenin escapes degradation, accumulates in the cytoplasm, and translocates to the nucleus, where it drives transcriptional activation of Wnt target genes, ultimately promoting tumorigenesis (12, 13).

**Figure 1:**
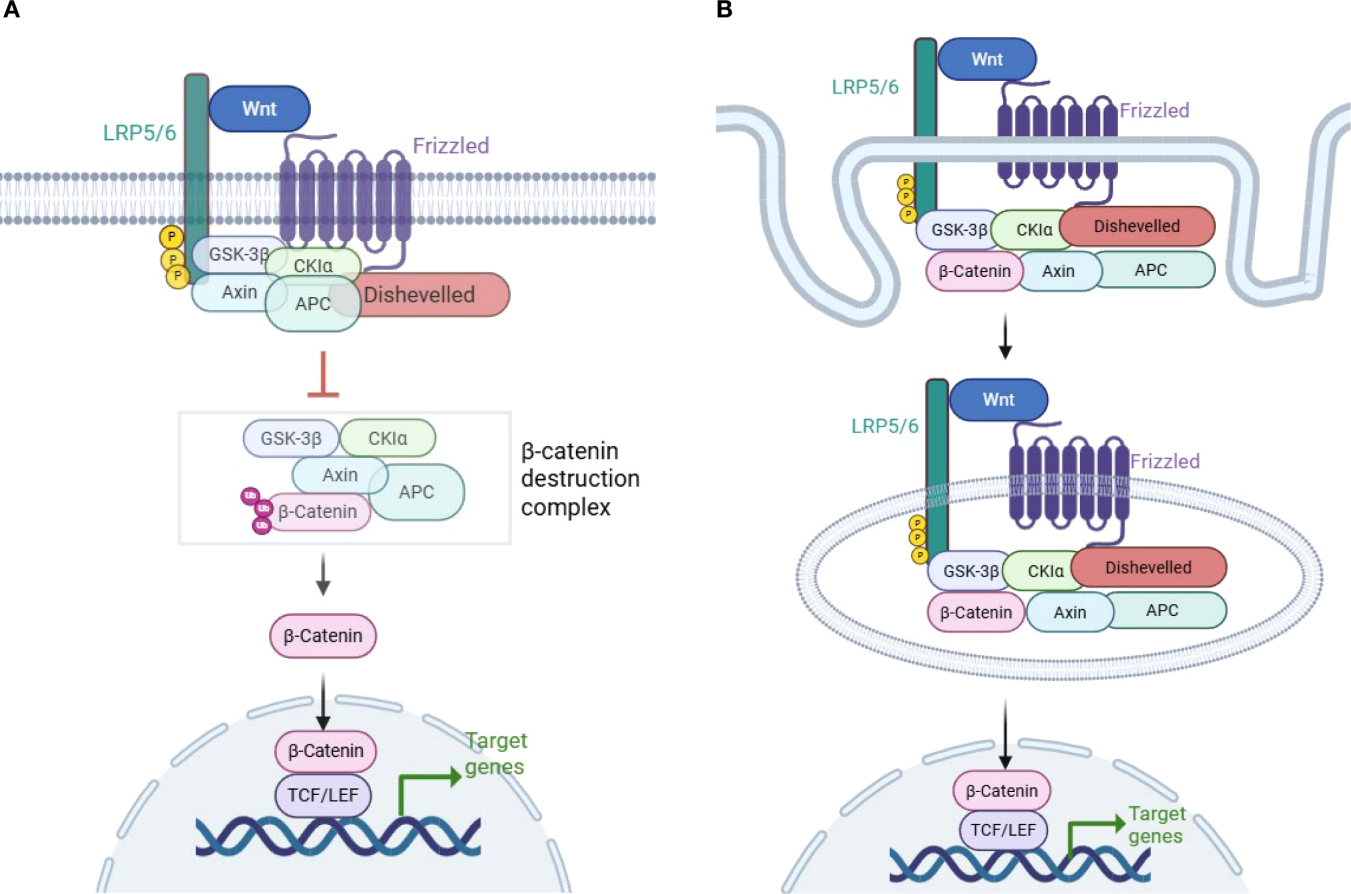
Wnt/β-catenin signaling pathway activation. **(A)** Model of LRP6 phosphorylation and DVL disruption of the β-catenin “destruction complex”. Wnt/β-catenin signaling is activated when Wnt binds to the Frizzled (FZD) and lipoprotein-related protein 5 or 6 (LRP5/6) coreceptors. Subsequent phosphorylation of LRP6 leads to the recruitment of Axin1 and Dishevelled (DVL). This process disrupts the β-catenin destruction complex, which is composed of GSK3β, CK1α, APC, and Axin1. As a result, β-catenin is stabilized in the cytoplasm and translocates to the nucleus, where it promotes the transcription of target genes (4–6, 10, 11). **(B)** Model of β-catenin inclusion in multivesicular bodies (MVBs) and escape from proteasomal degradation. A different model of Wnt/β-catenin signaling pathway activation has been proposed, in which the formation of MVBs following the endocytosis of the Wnt receptor encapsulates β-catenin along with the Dvl–GSK3β–CK1α–APC–Axin1 complex in MVBs. This process physically separates β-catenin from its cytoplasmic substrates, thereby preventing its degradation and facilitating its translocation into the nucleus (15–17) (created with Biorender.com).

In the canonical Wnt signaling pathway activation, Wnt ligands initiate the cascade by binding to Frizzled (FZD) receptors and co-receptors lipoprotein-related protein 5 or 6 (LRP5/6), which promote LRP6 phosphorylation and subsequently recruit Axin and activate Dishevelled (DVL) (11, 14).This process prevents the activation of the “destruction complex”, and upregulates of β-catenin in the cytoplasm. Consequently, β-catenin translocates into the nucleus and combines with TCF/ LEF, promoting the transcription of target genes (**Figure 1A**) (4–7, 10, 11, 14, 18).

There is another model of Wnt/β-catenin signaling activation. The multivesicular bodies (MVBs) model suggests that after Wnt binds to its receptor and recruits the β-catenin complex, Wnt and its associated receptors are endocytosed to form MVBs. During this process, endocytosis of the Wnt receptor encapsulates the DVL–GSK3β–CK1α–APC–Axin1 complex along with β-catenin, which physically separates β-catenin from its cytoplasmic substrates, preventing β-catenin phosphorylation and facilitating its translocation into the nucleus (**Figure 1B**) (15–17). The MVBs model of β-catenin activation has long been discussed and has been demonstrated in some cell lines.Tejeda-Muñoz N et al. found that macropinocytosis could be induced by Wnt-stimulating agents, such as the overexpression of DVL and FZD8 (19). In addition, they reported that the addition of Wnt3a to several cell lines stimulated the formation of large MVBs, which sequestered cytosolic GSK3 (20).The findings suggest that the two models of Wnt/β-catenin signaling activation may imply different physiological statuses: acute versus chronic status (16).

Another view suggests that β-catenin associates with E-cadherin to establish cell–cell adhesion. Epidermal growth factor receptor (EGFR), Met, and other molecules phosphorylate β-catenin, leading to its dissociation from cell–cell adhesion. This dissociation promotes the translocation of β-catenin into the nucleus, thereby increasing its transcriptional activity (4, 9, 11).

The association of β-catenin with E-cadherin at cell–cell adhesion may also play a role in the Wnt/β-catenin signaling pathway, which requires further investigation. Currently, considerable research has been performed on the role of nuclear β-catenin in promoting transcription; however, studies on membrane-associated β-catenin remain relatively scarce. Some studies have indicated that disrupting β-catenin cell–cell adhesion increases the motility and migratory ability of tumour cells (24). Additionally, it is important to explore whether other β-catenin-associated adhesion molecules, such as E-cadherin and γ-catenin, also modulate β-catenin activity within the Wnt signaling pathway in HCC (21–24).

**Figure 2:**
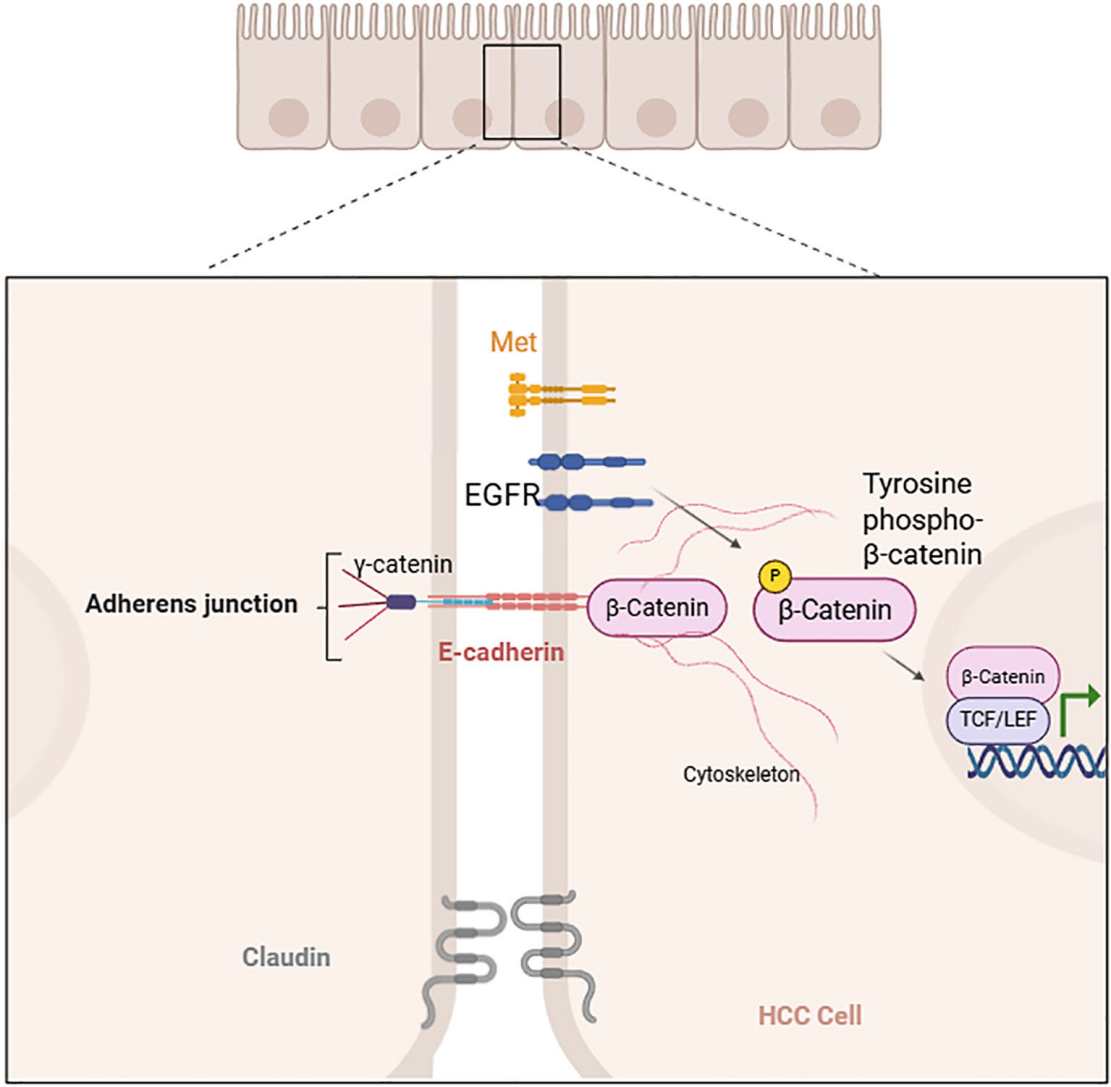
Tyrosine phosphorylation-induced dissociation of β-catenin from cell–cell adhesion (created with Biorender.com).

In a normal liver, β-catenin functions as a connector between the intracytoplasmic tail of E-cadherin, γ-catenin, and the actin cytoskeleton to regulate cell adhesion. In HCC, epidermal growth factor receptor (EGFR), Met, and other factors induce tyrosine phosphorylation of β-catenin, leading to its dissociation from cell–cell adhesion and and activation, which further promotes its entry into the nucleus to interact with TCF/LEF and influence gene transcription (**Figure 2**) (4, 9, 11, 23-24).

## 3 Wnt/β-catenin pathway in HCC development

The Wnt/β-catenin pathway is an evolutionarily conserved signaling pathway that control fundamental physiological and pathological processes. However, abnormal activation of the Wnt/β-catenin signaling pathway promotes HCC (4–7, 11). The activated Wnt/β-catenin signaling pathway synergizes with various signals to promote the proliferation of cancer stem cells (CSCs), drive HCC formation and facilitate tumour progression (25, 26). Therefore, a deeper understanding of the pathogenic factors causing abnormal Wnt/β-catenin signaling pathway activation in HCC will aid in the development of suitable drugs targeting this pathway for clinical treatment.

### 3.1 Morphological pathological characteristics of HCC with β-catenin mutation

The classic morphology of β-catenin-mutated HCC is characterized by well-differentiated tumours that feature tumour cells with abundant eosinophilic cytoplasm, thin trabeculae (≤ 2 cell layers), pseudoglands, and bile production. However, only 58% of cases with CTNNB1 mutations alone fully conformed to this morphology, indicating significant heterogeneity. Clinical studies have identified classic CTNNB1-mutated tumours as “immune cold,” which exhibit no response to checkpoint inhibitor therapies.Torbenson M et al. reported at least five distinct CTNNB1-specific missense mutations: S33P (4/5), D32V (3/5), T41A (2/5), D32G (2/7) and N387 (1/5); these mutations are associated with the classic morphology of β-catenin-mutated HCC (27).

Rebouissou S et al. reported that specific mutations may be related to increased levels of β-catenin activation and the malignant transformation of hepatocellular adenoma (HA) to HCC: (1) Mutations at the β-TRCP binding site (D32-S37) and deletions in exon 3 were linked to high activity and an elevated risk of malignant transformation. (2) T41 mutation is relative to moderate activation of β-catenin. (3) The S45 mutation is a rather special case. (4) K335 and N387 mutations resulted in weak activation and are likely associated with a low risk of malignant transformation to HCC (28).

Mutations within exon 3, particularly deletions and point mutations on residues D32-S37, disrupt the β-catenin “destruction complex” and impair the binding site for ​​β-TrCP​​, thereby suppressing β-catenin ubiquitination and degradation. This disruption leads to the accumulation of β-catenin and strong β-catenin activity (28).

In the β-catenin “destruction complex” model, residues ​​S33, S37 and T41​​ serve as phosphorylation sites for GSK-3β (12, 13), which play a critical role in β-catenin phosphorylation and degradation. Point mutations at ​​T41​​ typically result in ​​moderate activity​​ of β-catenin, a level observed in both benign and malignant tumors. Moderate-to-high levels of β-catenin are necessary for malignant progression. The moderate activity of T41 point mutations provides the “just right” signaling model in both tumor types (28).

S45 (phosphorylation sites for CK1α) mutations are complex (12, 13), exhibiting weak activation of β-catenin in HA. However, most S45 mutant alleles in HCC are duplicated and cooperate with a second oncogenic signal, resulting in a high activation of β-catenin. The roles of residues K335 and N387 in regulating weak β-catenin activity are not yet fully understood and need further investigation (28).

CTNNB1 mutation in hepatocellular tumors activates Wnt/β-catenin and overexpresses classical β-catenin target genes such as GLUL (coding for glutamine synthase, GS). Immunohistochemistry (IHC) staining of GS was related to with β-catenin activity in hepatocellular tumors. Strong and diffuse GS staining was associated with increased β-catenin mutation activity and tumour malignancy, which was observed in overt HCC as well as in lesions with borderline features between HA and HCC. In contrast, weak β-catenin mutations, such as K335 and N387 mutations, were demonstrated by faint GS staining and were more frequently found in benign HA at low risk of malignant transformation (28).

In HA, β-catenin mutations may upregulate the GS gene expression, further promoting β-catenin activity. This can lead to the malignant transformation of the HA subtype to HCC, as indicated by abnormal nuclear staining of β-catenin and strong immunohistochemical staining of GS. However, GS overexpression should not be considered the defining characteristic of increased β-catenin activity, and many cases that do not exhibit atypical morphologic or clinical signs of HCC may show strong GS staining (29).In contrast, the combined use of other tissue markers such as GPC3, HSP70, glypican-3, and CD10 can further increase specificity and sensitivity (30).

Currently, several tissue markers related to increase β-catenin activity are available for studying HCC. For example, CD10, a membrane zinc-dependent metalloproteinase, is overexpressed in relation to the aggressiveness of human cancers, particularly in HCC (30). Kim HS reported that stromal CD10 expression correlated with cytoplasmic β-catenin accumulation. They speculated that CD10, which is secreted by stromal cells, may induce other proteases or matrix metalloproteinases (MMPs), cleaving E-cadherin at the cancer cell membrane, sequestering E-cadherin from the membrane with β-catenin, and leading to cytoplasmic β-catenin accumulation. However, this study focused on breast carcinoma, and the mechanisms underlying the dysregulation of CD10 and β-catenin in HCC need further investigation (31).

### 3.2 The molecular mechanisms by which the Wnt/β-catenin pathway regulates HCC

Most HCC cases have mutations in genes that regulate the Wnt/β-catenin pathway. Research has shown that CTNNB1 mutations occur in 25% of HCC cases in mouse models, whereas in HCC patients, the CTNNB1 mutation rate is approximately 11%~40% (4, 32, 33). Most CTNNB1 gene showed missense point mutation in exon 3, and the frequency of the point mutation was significantly higher in non-viral HCCs (29.4%) rather than HBV-related cases (12.7%) (34).

Torbenson M et al. analyzed data from The Cancer Genome Atlas (TCGA) and reported that among 338 cases (7 with fibrolamellar carcinomas and 331 with conventional HCC), 128 cases had mutations in the CTNNB1, APC, and Axin genes (38%). Among these, 88 cases had CTNNB1 mutations alone (26%), 4 cases had both CTNNB1 and APC mutations, 2 cases had CTNNB1 and Axin mutations, 26 cases had Axin mutations alone (8%), 7 cases had APC mutations alone (2%), and 1 case had both Axin and APC mutations (27).

The CTNNB1 mutation rate was significantly more frequent in males than in females (35% vs. 13%). In contrast, the mutation rates showed no significant gender difference in Axin (8% vs. 10%) and APC (4% vs. 2%) mutations (27).

Other Studies also have shown that APC mutations occur in 3% of HCC patients (35–39). Furthermore, loss-of-function (LOF) mutations in Axin1 have been identified in approximately 3–16% of HCC patients, whereas Axin2 mutations are present in approximately 3% of HCC patients (40–43). Additionally, loss of function of GSK3β has been reported in HCC (44). APC, Axin and GSK3β are primary components of the β-catenin “destruction complex”. These mutations lead to missense and nonsense mutations, resulting in “destruction complex” dysfunction and β-catenin accumulation in the cytoplasm (4).

In HCC, overexpression of the Wnt/β-catenin pathway components FZD7 and Wnt3 has been observed, leading to activation of this pathway (45). Additionally, methylation of the secreted frizzled-related protein genes sFRP1 and sFRP5 (46–48), TGF-β-dependent β-catenin activation, and activation of β-catenin by receptor tyrosine kinases have also been observed in HCC patients (49–51).

Although the Wnt/β-catenin pathway is activated in HCC, its activation alone is insufficient for liver tumour formation, likely due to the disease’s heterogeneity. Instead, a second oncogenic signal is required for the development of HCC (**Table 1**) (52–70). Mouse models that overexpress wild-type or stable mutant β-catenin do not develop HCC. However, β-catenin functions simultaneously with other signaling pathways to promote HCC formation and development (18). For example, neither activated β-catenin nor overexpression of c-Met alone promotes HCC formation; however, the combination of activated β-catenin co-expressed with c-Met via hydrodynamic transfection enhances HCC occurrence and progression in mice (52, 53). In HCC patients, concurrent activation of c-Met and β-catenin gene mutations was observed in 9%–12.5% of samples; additionally, co-activation of c-Met and Axin1 loss-of-function mutations was found in approximately 3%–5% of samples. The deletion of the Axin1 gene (sgAxin1) using CRISPR/Cas9 in mice—which stabilizes β-catenin—does not lead to HCC formation on its own; however, it cooperates with c-Met overexpression to promote HCC development. In contrast, co-expression of c-Met and sgAxin1 in liver-specific Ctnnb1 null mice did not promote HCC development. This further demonstrates that Axin1 deletion cooperates with c-Met expression to induce HCC in mice in a manner dependent on the β-catenin signaling pathway (66). Also, TP53 mutations were rare found in the typical morphology of CTNNB1-mutated HCC. TP53 mutations co-occurring with specific CTNNB1 mutations show clonal progression and multiple distinct morphologies (27).

**Table 1:** Signaling molecules that cooperate with β-catenin in HCC development.

Cooperation	Character	Reference
GS	Associated with malignancy	(28)
TGF-β	Promoted tumour proliferation, diffusion and metastasis	(50, 51)
c-Met	Induced tumour formation and development	(52, 53)
RAS	Promoted tumour cell proliferation and viability	(54)
EGFR	Promoted HCC metastasis	(55)
MYC	Associated with poor prognosis	(56)
P53	Related to HCV, HBV virus infections in HCC development	(27, 57, 58)
Akt	Formed fatty hepatocellular adenomas that progressed to HCC	(59)
LKB1	Conferred a better prognosis and a well-differentiated growth pattern	(60)
Nrf2	Led to HCC development	(61, 62)
TERT	Related to HCV, HBV, HCC	(63)
m^6^A	Upregulation of FZD10-liver cancer stem cell properties and lenvatinib resistance	(64)
FOXM1	Promoted metabolic reprogramming, angiogenesis, and the maintenance of cancer stem cell properties	(65)
Glypican-3	Promoted the growth of HCC	(66–70)

Interestingly, interactions between the activity of glypican-3 (GPC3) and Wnt/β-catenin have also been reported to induce HCC. GPC3 not only serves as a diagnostic and prognostic biomarker but also plays a functional role in activating the Wnt/β-catenin signaling pathway. Advanced HCC is characterized by high levels of GPC3 and the FZD receptor, whereas healthy liver tissue typically has low concentrations of both (67). GPC3 is associated with the Wnt/β-catenin signaling pathway to promote HCC formation and development (68–70). Lai JP et al. reported that cell surface glypican-3 (GPC3) stimulates Wnt/β-catenin signaling in HCC by forming a complex with Wnt ligands via its heparan sulfate glycosaminoglycan (HSGAG) chains. Sulfatase 2 (SULF2)—an enzyme often overexpressed in HCC—removes 6-O-sulfate groups from HSGAG, disrupting this complex, releasing Wnt, and leading to enhanced Wnt signaling activity (68). Targeting this pathway, Wei Gao et al. demonstrated that an immunotoxin against GPC3 inhibits both Wnt signaling and protein synthesis in HCC cells, resulting in tumor regression (69). Additionally, Dan Li et al. found that chimeric antigen receptor (CAR) T cells targeting GPC3 effectively eliminate GPC3-positive HCC cells in mice with xenograft or orthoptic liver tumors through mechanisms such as reduction of Wnt signaling or induction of perforin/granzyme-mediated apoptosis (70).

### 3.3 Characteristics of HCC with Wnt/β−catenin pathway activation

Abnormal activation of the Wnt/β-catenin signaling pathway in patients with HCC results in unique clinical and pathological features. The rate of CTNNB1 mutation is influenced by the etiology of HCC. Several studies have shown a high rate of CTNNB1 mutations in HCC associated with hepatitis C virus (HCV) infection, with over 40% of tumours presenting mutations (4, 71). However, findings regarding CTNNB1 mutations in HBV-related HCC are limited, suggesting that HBV may activate the Wnt/β-catenin signaling pathway through alternative mechanisms (34).

Although β-catenin activation of the Wnt pathway has been observed in liver cirrhosis tissues infected with HBV, studies have shown that the frequency of CTNNB1 gene mutations is not related to HBV infection. HBV may activate the Wnt pathway through epigenetic changes associated with HBV-related HCC or may cause dysregulation of the Wnt pathway due to effects on the TCF/β-catenin transcription. A study by Amaddeo G, et al. found that HBV-related HCCs exhibit significant genomic diversity and can belong to all transcriptomic subgroups. HBV infection may lead to TP53 mutations, overexpression of stem cell genes, and impaired cell reprogramming associated with HBV-related HCC (72). Tran BM, et al. also reported that the HBV pre-core protein p22 elevated Wnt signaling by activating TCF/β-catenin transcription, which drives liver cancer (73). Additionally, Wang MH et al. reported that Musashi-2 (MSI2), a member of the Musashi family, upregulated β-catenin and TCF-4/LEF-1 expression, promoting hepatitis B virus-related HCC progression via the Wnt/β-catenin pathway (74). Studies also have shown that in liver cirrhosis tissues associated with HBV infection, the expression of the β-catenin and c-Myc genes is upregulated. Furthermore, β-catenin mutations at phosphorylation sites and their adjacent locations are associated with increased Wnt pathway activity, which may promote the occurrence and development of HBV-related hepatocellular carcinoma (HBV-HCC) (34).

HCC can be categorized into 2 main types: proliferative and non-proliferative. Each accounts for approximately 50% of the cases (4, 7). Multiple subtypes are further classified into these two categories. The proliferative type is the most prevalent type among patients infected with HBV and is characterized clinically by elevated serum levels of alpha-fetoprotein (AFP) (75), loss-of-function (LOF) mutations in TP53, chromosomal instability, high vascular invasiveness, and a poor prognosis. In contrast, the non-proliferative type is associated with HCV infection, presenting clinically with low AFP levels, short telomeres, low vascular invasiveness, hepatocellular differentiation, and good prognosis (4, 7, 76).

The proliferative type HCC is further subdivided into two subtypes: a “Wnt/TGF-β subtype” and a “progenitor subtype.” The “Wnt/TGF-β subtype” characterized by activation of the Wnt pathway that synergizes with TGF-β, shows immune exhaustion. The “progenitor subtype” is defined by overexpression of hepatic progenitor markers, hyperphosphorylation of extracellular signal-regulated kinases (ERK), inactivating mutations in Axin1and ribosomal protein S6 kinase A3 (RPS6KA3) (7). However, the “non-proliferative type” of HCC is more heterogeneous and includes CTNNB1 mutations. The Wnt/β-catenin pathway is active in both proliferative and non-proliferative HCC types, however, it affects different HCC phenotypes. Wnt synergizes with TGF-β, and Axin1 mutations are associated with the proliferative type, whereas CTNNB1 mutations are associated with the non-proliferative type. These findings suggest that activation of downstream pathways is more important than activation of the Wnt/β-catenin pathway alone (4, 66, 76, 77).

Kitao et al. reported that HCC exhibiting β-catenin CTNNB1 mutations displays iso-high intensity in the hepatobiliary phase (HBP) of gadolinium ethoxybenzyl diethylenetriaminepentaacetic acid-enhanced magnetic resonance imaging (Gd-EOB-DTPA-MRI) (78, 79), and HCC patients with CTNNB1 mutations have a better prognosis compared with patients with other types of HCC (80, 81). However, some studies have suggested that CTNNB1 mutations may not be associated with prognosis in patients with advanced HCC (82). Prognosis is a complex characteristic of HCC and is influenced not only by tumour factors, such as vascular invasion, metastasis, and the tumour microenvironment, but also by non tumour factors, such as fibrsis, cirrhosis, liver dysfunction, and extrinsic factors. Therefore, more research is needed to determine whether CTNNB1 mutations and Wnt/β-catenin activation affect tumour phenotypes and patient prognosis (83–87).

### 3.4 The role of the Wnt/β-catenin pathway in immunotherapy of HCC

HCC can be categorized into three classes on the basis of immune status: “immune class”, “immune exclusion class”, and “immune intermediate class”, each accounting for approximately 30% of the cases (78, 88). The “immune class” is characterized by high levels of immune cell infiltration and is more likely to respond to immune checkpoint inhibitor (ICI) therapy. On the other hand, the “immune exclusion class” is characterized by T-cell exclusion from the tumour microenvironment (TME) and CTNNB1 mutations, resulting in resistance to ICI therapy. The “immune intermediate class”, which has wild-type CTNNB1 and intermediate levels of immune infiltration, requires further characterization to predict the response to immunotherapies (78, 88).

**Figure 3:**
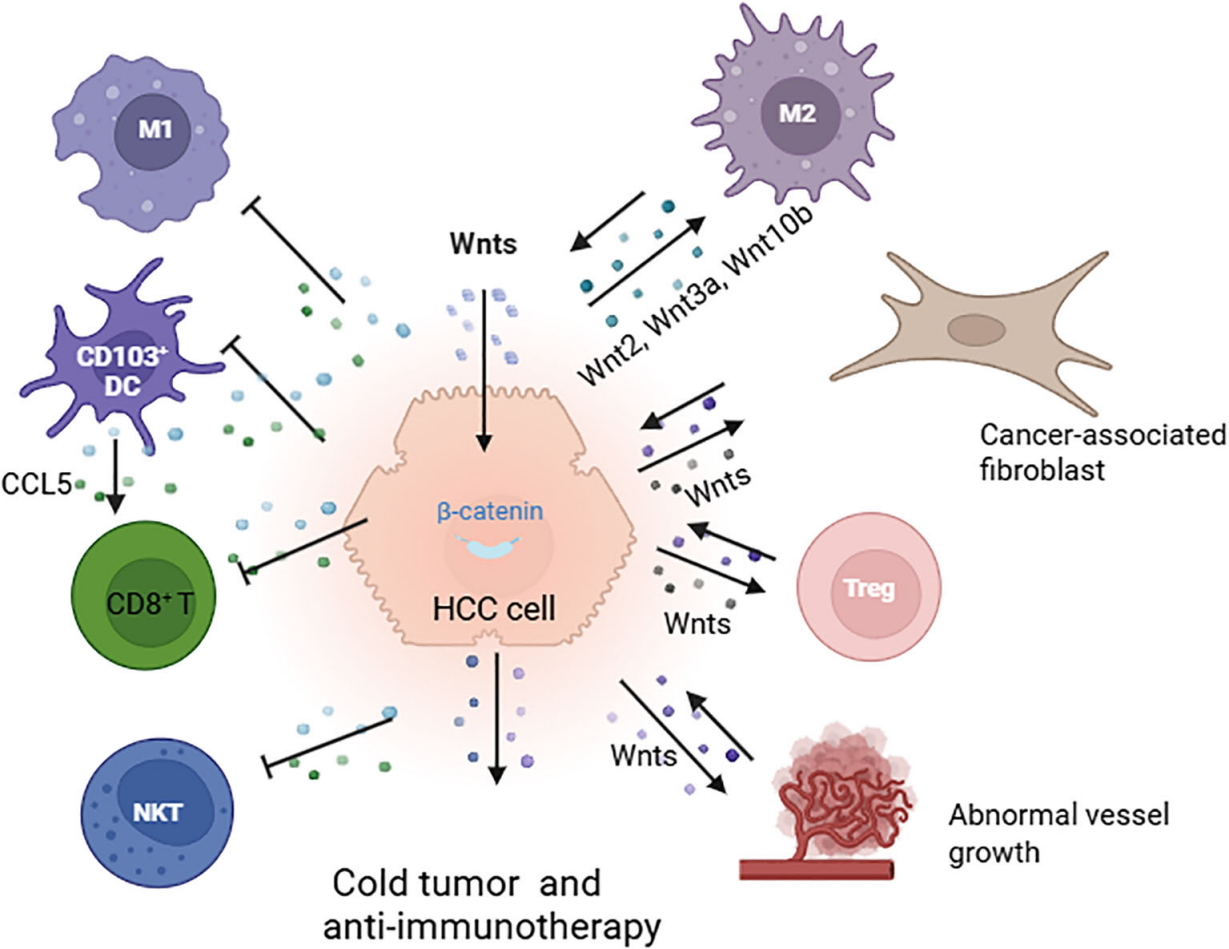
Immune exclusion mediated by the Wnt/β-catenin pathway in the tumour microenvironment (TME). Activation of Wnt/β-catenin signaling facilitates cross-communication between cancer cells and various cells in the TME, leading to cold tumours and resistance to immunotherapy. This process involves the polarization of M2-type macrophages through Wnt2, Wnt3a, and Wnt10b; interaction with fibroblasts; enhancement of Treg survival; promotion of abnormal vessel growth; reduction in the recruitment of CD103^+^ dendritic cells; decreased CCL5 production; and diminished infiltration of CD8^+^ T cells and NKT cells (78, 88, 89)(created with Biorender.com).

CTNNB1 mutations and Wnt/β-catenin pathway activation are closely associated with the “immune exclusion class” that is characterized by immune exclusion and anti-immunotherapies in HCC (**Figure 3**) (78, 88, 89). The Wnt/β-catenin signaling pathway promotes communication between cancer cells and various cells in the TME, such as fibroblasts, endothelial cells, and lymphocytes. This communication facilitates tumour angiogenesis, metastasis, and immune escape (89). Therefore, developing drugs that target the Wnt/β-catenin signaling pathway in the TME can increase the efficacy of immunotherapy (89–91).

In mouse models of HCC, Wnt ligands, such as Wnt2, Wnt3a, and Wnt10b, produced by liver tumour cells activate the Wnt/β-catenin pathway. This activation leads to the proliferation of tumour-associated macrophages (TAMs) and polarization of M2-type macrophages, thereby promoting tumour growth and metastasis (90). Additionally, the Wnt signaling pathway plays a significant role in thymocyte development, including the polarization, differentiation, and maturation of thymic T cells. For example, Wnt proteins secreted by thymic epithelial cells support thymic development and the stabilization of regulatory T cells (91, 92). The Wnt signaling pathway also enhances the survival of immunosuppressive Treg cells while inhibiting the expansion of CD8^+^ T cells (**Figure 3**) (93, 94).

Furthermore, studies in mouse models of HCC have shown that β-catenin activation inhibits the recruitment of dendritic cells, resulting in reduced CD8^+^T-cell activity and the promotion of immune escape. Preclinical research indicates that tumours with activated β-catenin exhibit resistance to immune checkpoint therapy that targets PD-1 (95). Moreover, inhibiting the Wnt signaling pathway in the TME can increase the activity of natural killer T cells and promote the secretion of interferon-gamma (IFN-γ) (96, 97).

CTNNB1 gain-of-function (GOF) mutations promote MMP9 secretion in the TME of HCC, inhibiting the activity of CD8^+^ T cells and leading to the immune escape of tumour cells and resistance to anti-PD-1 therapy. Targeting MMP9 can restore the TME and enhance the effectiveness of anti-PD-1 treatment (98). Additionally, by inhibiting the Wnt/β-catenin signaling pathway, the expression of the chemokine CCL5 can be upregulated, which recruits dendritic cells (DCs) into the TME. This results in increased infiltration of CD8^+^ T cells into the TME, enhancing the antitumour immune response (99). Furthermore, by targeting both β-catenin and PD-L1 with a racemic supramolecular peptide, it is possible to increase the infiltration of CD8^+^ T cells at the tumour site, thereby increasing the efficacy of immunotherapy for HCC (100).

M. Kudo et al. reported that HCC with Wnt/β-catenin mutations is resistant to ICI therapy. In a clinical study, 10 patients with Wnt/β-catenin mutations did not respond to ICI therapy. In contrast, a complete response was achieved in patients without Wnt/β-catenin mutations. Progression-free survival (PFS) was shorter in patients with Wnt/β-catenin mutations compared to those without mutations (2 months vs. 7.4 months). Similarly, overall survival (OS) was also lower in patients with Wnt/β-catenin mutations than in those without (9.1 months vs. 15.2 months). Although this study included a small number of cases, it represents a significant breakthrough, providing clinical evidence for the hypothesis that HCC with Wnt/β-catenin mutation/activation behaves as an immune-cold tumour. This is due to the reduced infiltration of CD8^+^ T cells, which leads to resistance to ICI therapy (78, 101).

Most interestingly, the Wnt/β-catenin mutation did not affect the treatment efficacy of sorafenib (78, 101, 102). These findings suggest that the Wnt/β-catenin mutation status may not impact the efficacy of tyrosine kinase inhibitors (TKIs) such as lenvatinib, ramucirumab, cabozantinib, and regorafenib. However, it remains uncertain whether combination therapies, such as ICI combined with TKI therapy, are effective in HCC patients with Wnt/β-catenin mutations. Some studies have shown that combination therapies have favorable results, characterized by low progression disease (PD) rates. In combination trials of HCC, the PD rates for pembrolizumab plus lenvatinib were significantly lower at 7% compared with 32.4% for pembrolizumab monotherapy (78, 101, 102). These positive results, characterized by low PD rates in combination immunotherapy, may largely be due to the additive anticancer effect of lenvatinib, even in patients with HCC with Wnt/β-catenin mutations (78, 101, 102).

The Wnt signaling pathway inhibits CD8^+^ T-cell activity in the TME, promoting tumour immune escape and resistance to ICI therapy. However, several studies have suggested that the Wnt signaling pathway may also promote antitumour effects in the TME. For example, the Wnt pathway can stimulate the maturation of natural killer (NK) cells and induce the generation of CD8^+^ memory stem cells. Additionally, the Wnt signaling pathway promotes the survival of B cells and helps reduce the number of myeloid-derived suppressor cells (MDSCs) in the TME (89, 103, 104).

## 4 Targeting the Wnt/β−catenin pathway for HCC therapy

**Figure 4:**
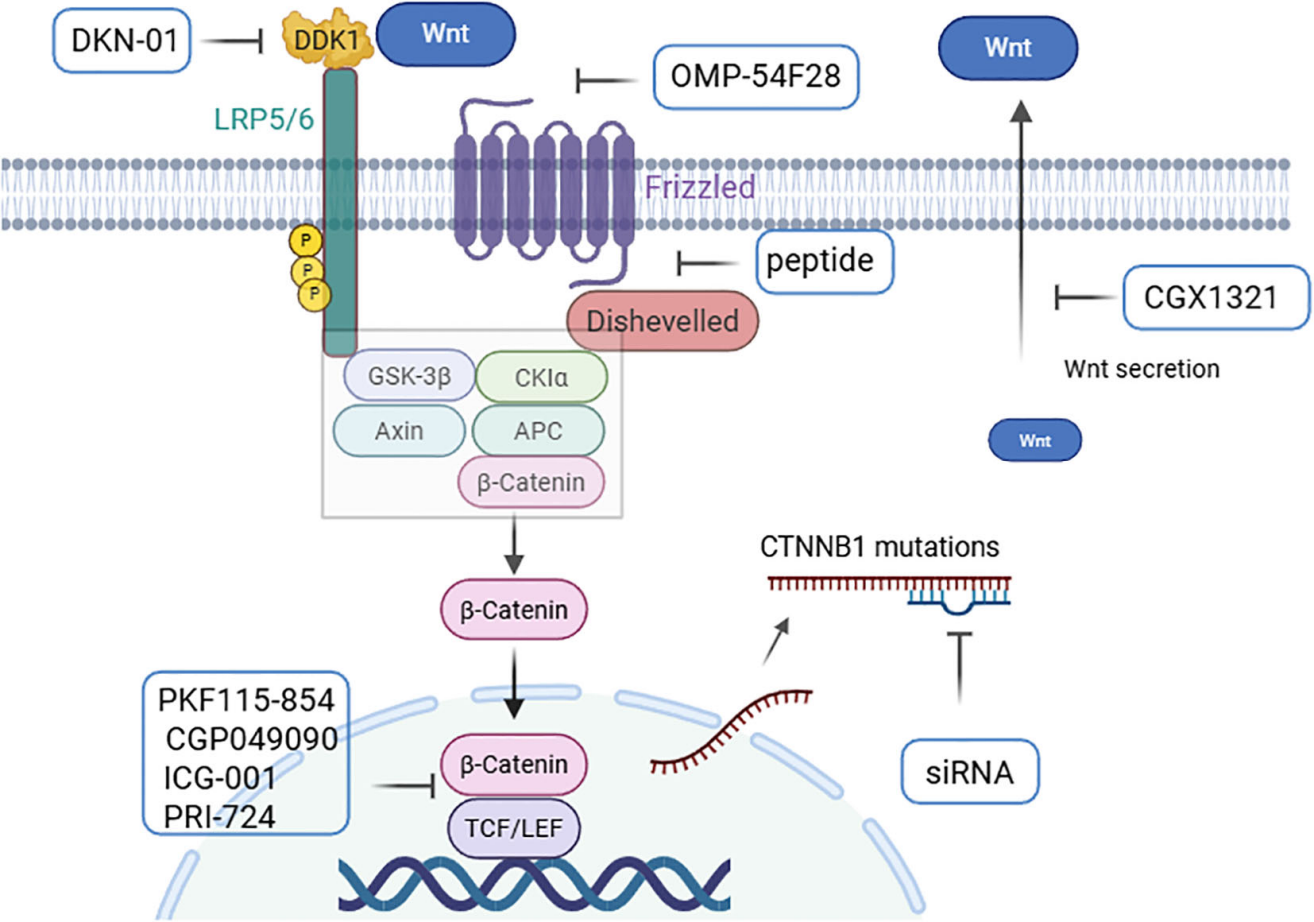
Pharmacological regulators based on Wnt/β-catenin in HCC. Regulation of the Wnt signaling pathway by (1) targeting Wnt ligands and receptors (CGX1321, OMP-54F28, and DKN-01); (2) stabilizing the β-catenin “destruction complex” with peptides; (3) targeting CTNNB1 mutations with siRNAs; and (4) preventing β-catenin transactivity in the nucleus (PKF115-854, CGP049090, ICG-001, and PRI-724) (4–6) (created with Biorender.com).

Recently, various therapeutic strategies targeting the Wnt/β-catenin pathway have been developed. These strategies include the use of monoclonal antibodies or small molecule inhibitors to target Wnt ligands and FZD and LRP5/6 receptors. Additionally, there are approaches aimed at stabilizing the β-catenin “destruction complex” to promote β-catenin degradation, targeting the CTNNB1 gene, and inhibiting the interaction between β-catenin and the nuclear transcriptional regulators (**Figure 4**) (4–7). Several agents targeting the Wnt/β-catenin pathway have been evaluated in clinical trials for the treatment of HCC or HCC-related diseases (**Table 2**) (3).

**Table 2:** Clinical trials evaluating Wnt/β-catenin-targeted agents in HCC or HCC-related diseases.

Agents	Mechanism	Phase	Side effects	Identifier
CGX1321(with pembrolizumab)	Porcupine inhibitor	Phase 1	NR	NCT02675946
OMP-54F28 (with sorafenib)	FZD8 decoy receptor	Phase 1	Diarrhea, neutropenia and decreased appetite	NCT02069145
DKN-01	DKK1	Phase 1	NR	NCT02375880
PRI-724	CBP/β-catenin antagonist	Phase 1	Nausea, vomiting, diarrhea, alopecia, fatigue, neutropenia, thrombocytopenia, neutropenic fever	NCT02195440

The use of monoclonal antibodies or small molecules to inhibit Wnt ligands and receptors can enhance apoptosis and suppress cell proliferation. Porcupine, an acyltransferase, facilitates the acylation of Wnt proteins by providing palmitoyl groups, which enhances Wnt secretion. The small molecule inhibitor CGX1321 targets porcupine activity, thereby preventing Wnt secretion (105, 106). CGX1321 has been evaluated in a phase I clinical trial (NCT02675946) for advanced solid tumours, including HCC and cholangiocarcinoma (6).

Owing to the upregulation of FZD expression in more than 60% of HCC cells, FZD is considered an important therapeutic target. FZD7 inhibition significantly promoted HCC cell apoptosis. Research has shown that soluble FZD7 (sFZD7) and monoclonal antibodies targeting FZD7 can inhibit FZD receptor activity, resulting in tumour growth suppression (107, 108). Additionally, studies have shown that a fusion protein, OMP-54F28, can compete with its ligands for the FZD8 receptor and antagonize the Wnt signaling pathway, thereby inhibiting the growth of liver cancer, ovarian cancer, and pancreatic cancer. OMP-54F28 was tested in a phase I clinical trial (NCT02069145) (6).

DKK1(Dickkopf-1) is an antagonist of the Wnt/β-catenin signaling pathway that inhibits the activation of the pathway by interacting with LRP5/6 and preventing Wnt from binding to the receptor. DKK1 has diverse effects under various physiological conditions, reflecting the complexity of the Wnt/β-catenin signaling pathway. Studies have demonstrated that DKK1 expression is often elevated in HCC and cholangiocarcinoma, promoting tumour proliferation, migration, and invasion (109–111). Furthermore, the monoclonal antibody DKN-01, which targets DKK1, was evaluated in combination with gemcitabine and cisplatin in a phase I clinical trial in patients with HCC and cholangiocarcinoma (NCT02375880) (6).

Small molecule inhibitors that block the interaction between β-catenin and TCF or other related cofactors or components of transcription complexes are promising targets for the treatment of HCC. Several small-molecule inhibitors, including PKF115–854 and CGP049090, can block the interaction between β-catenin and TCF, thereby inhibiting HCC growth *in vivo* (112, 113).To form a transcriptionally active β-catenin/TCF complex, β-catenin recruits transcriptional coactivators, such as cyclic AMP response element-binding protein (CBP), along with other components of the basic transcription machinery. The CBP/β-catenin antagonist ICG-001 and its active enantiomer PRI-724 which antagonize β-catenin/TCF-mediated transcription and specifically eliminate tumour stem cells (114, 115). PRI-724 was evaluated in clinical trial to treat HCV cirrhosis (NCT02195440) (116).

Stabilizing the β-catenin destruction complex and promoting β-catenin degradation are effective strategies for inhibiting the Wnt/β-catenin pathway. When small-molecule peptides are used to block the interaction between FZD7 and DVL, the “destruction complex” can be stabilized, β-catenin degradation can be increased, thereby suppressing HCC cell growth (117). Additionally, targeting CTNNB1 with nucleic acid medicines, such as antisense oligonucleotides (ASOs) and small interfering RNAs (siRNAs), represents a promising approach. Studies have shown that CTNNB1 mutations can activate the Wnt/β-catenin pathway and drive HCC development. ASOs designed to target CTNNB1 mutations can slow HCC progression in murine models (118). Furthermore, a strategy that utilizes lipid nanoparticles (LNPs) combined with Dicer-substrate siRNA targeting CTNNB1 significantly inhibited liver tumour development *in vivo* (119).

Although the Wnt/β-catenin pathway is essential for maintaining homeostasis in normal tissues, significant concerns remain regarding the toxicity of inhibitors that target Wnt signaling pathways. Therefore, identifying molecules that are more specific to HCC is a promising strategy for targeting the Wnt/β-catenin pathway. Research has shown that ADP-ribosylation factor (ARF)-like 4c (Arl4c) expression is stimulated by the activation of the β-catenin or EGFR-MAP kinase pathways, contributing to tumourigenesis in various cancer types, including HCC. Antisense oligonucleotides targeting Arl4c effectively inhibited HCC development *in vivo* (102).

## 4 Conclusion and perspectives

The Wnt/β-catenin signaling pathway plays crucial roles not only in the physiological functions of the normal liver but also in the development of HCC (4, 5). The Wnt/β-catenin pathway has distinct gene expression profiles and pathological characteristics in HCC, making it a promising therapeutic target (6, 7). Monoclonal antibodies or small molecule inhibitors that target key regulatory factors, such as Wnt ligands, FZD receptors, DVL, and CTNNB1 mutations, or inhibit the interaction between β-catenin and the nuclear transcriptional regulators can partially or completely shut down the Wnt/β-catenin signaling pathway, thereby suppressing tumour growth. Several small-molecule drugs and antibodies have advanced into clinical trials and have shown promising results.

Although some trials have been halted because of limited compound supply or insufficient patient recruitment, no clinical trials have ever been terminated because of a lack of drug efficacy. Ongoing clinical trials will be completed in the coming years and analyzed. Given the intrinsic heterogeneity of cancer, this suggests that combination therapy involving Wnt/β-catenin pathway-targeted drugs and anticancer medications could lead to favorable outcomes and FDA approval (120). In recent years, interest in the role of the Wnt/β-catenin pathway in regulating the TME and immune evasion in HCC has increased. Research has also focused on inhibiting the activity of the Wnt/β-catenin pathway to increase the effectiveness of immunotherapy (4–7, 89).

Despite extensive research on this pathway in HCC development, our understanding of its dysregulation remains limited. Given that the Wnt/β-catenin pathway plays a crucial role in normal tissue as well as in liver homeostasis and regeneration, systemic inhibition of factors within this pathway during treatment may lead to severe side effects. The off-target effects of the Wnt/β-catenin signaling pathway include nausea, vomiting, diarrhea, kidney damage, bone toxicity, and intestinal toxicity. Therefore, ensuring the safety and selectivity of targeted drugs is particularly important (121–124).

Furthermore, the Wnt/β-catenin pathway may participate in signal transduction pathways and have multiple homologs at different levels, which may lead to redundancy and adaptability in individuals with specific gene deletions. Additionally, research on the interactions between the Wnt/β-catenin signaling pathway and other signaling pathways remains lacking, resulting in findings that contradict those of other studies. This complicates the assessment of the therapeutic effects of interventions in the Wnt/β-catenin signaling pathway (1, 120).

Therefore, targeting HCC-specific Wnt/β-catenin pathway genes is crucial for therapy. This pathway has also been shown to be an important factor in tumour immune evasion and anti-immunotherapy. Consequently, identifying key molecules within this pathway to develop effective treatment strategies is a promising area of research (125–128). Regulating the Wnt/β-catenin pathway and activating the immune system is a strategy for treating HCC. The targeting of the Wnt/β-catenin pathway in combination with immunotherapy and the synergistic effects of TKIs will be clarified in future studies (78). In summary, a better understanding of the role of Wnt/β-catenin signaling in HCC will provide important strategies for effective treatment.

## Glossary

APC: adenomatous polyposis coli
Axin: axis inhibition protein
CK1α: casein kinase I isoform α
CBP: cyclic AMP response element-binding protein
Dkk: Dickkopf protein
DVL: Dishevelled
EGFR: epidermal growth factor receptor
ERK: extracellular signal-regulated kinases
FZD: Frizzled
FZD8: frizzled family receptor 8
FRPs: frizzled protein-related proteins
Gd-EOB-DTPA-MRI: gadolinium ethoxybenzyl diethylenetriaminepentaacetic acid-enhanced magnetic resonance imaging
GSK-3: glycogen synthase kinase-3
HBP: hepatobiliary phase
HCC: hepatocellular carcinoma
HIF1α: hypoxia-inducible factor 1α
HBV: chronic hepatitis B
HCV: hepatitis C virus
ICI: immune checkpoint inhibitor
LRP5/6: lipoprotein–related protein 5 or 6
LEF: lymphocyte enhancer factor
Met: Hepatocyte Growth Factor Receptor
RPS6KA3: ribosomal protein S6 kinase A3
sFZD7: soluble FZD7
TCF: T-cell factor
TKIs: tyrosine kinase inhibitors.
